# Necrotising Inflammation of Peri-Pancreatic Tissue With Normal Appearance of Pancreas Is a Distinct Entity of Acute Pancreatitis: Report of Two Cases With Review of Literature

**DOI:** 10.7759/cureus.43075

**Published:** 2023-08-07

**Authors:** Pagadala Nitesh, Pottakkat Biju, Raja Kalayarasan, Pothugunta S Krishna

**Affiliations:** 1 Surgical Gastroenterology, Jawaharlal Institute of Postgraduate Medical Education and Research, Puducherry, IND

**Keywords:** revised atlanta’s classification, necrotising pancreatitis, peri-pancreatic inflammation, acute pancreatitis, extrapancreatic necrotising pancreatitis

## Abstract

Improved insights into the pathophysiology of acute pancreatitis have paved the way for identification of distinct entities in the spectrum of the disease. The presence of necrotising inflammation limited to peripancreatic tissue with a normal appearance of pancreas is one such entity. This entity, described as extrapancreatic necrotising pancreatitis (EPN), is considered a less aggressive form of acute necrotising pancreatitis. This entity needs to be recognized precisely and managed accordingly among patients with acute pancreatitis. However, EPN has not been highlighted in the revised classification of acute pancreatitis. Here we report two patients with EPN with varied presentations and diverse management and outcome.

## Introduction

Acute pancreatitis, an acute inflammatory disorder of the pancreas, can have a varied spectrum of presentation ranging from mild acute pancreatitis to severe acute necrotising pancreatitis with organ failure. The intriguing nature of the course and outcomes in patients with acute pancreatitis is further complicated by different terminologies used in the description of this entity. This has necessitated a revision of the Atlanta classification of acute pancreatitis in 2012 by international consensus [1]. Better insights into the pathophysiology of acute pancreatitis translated not only to improved management of patients with the most severe form of the disease but also facilitated standardization in reporting of data. Some patients present with imaging features of either more than fat stranding or necrosis limited to peripancreatic tissues with an innocuous appearance of pancreas on imaging. This is considered a distinct entity of acute pancreatitis called extrapancreatic necrotising pancreatitis (EPN). However, the proposed pathophysiology of this entity is purely hypothetical and not conclusively described in the literature. In addition, based on the diversity in outcomes of patients with EPN, this entity needs to be addressed with lucidity in patient management. Here we report two patients with EPN to add further evidence to this novel entity.

## Case presentation

Case 1

**Figure 1:**
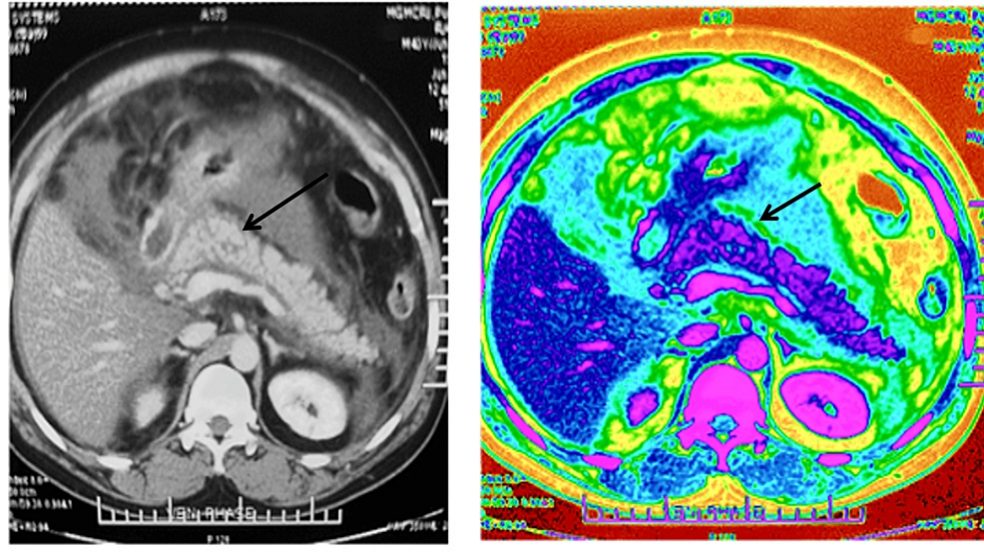
A: Contrast-enhanced computed tomography (CECT) image of patient in Case 1 showing normal enhancement of entire pancreas with preserved lobularity of the gland (black arrow). B: In the processed image of patient in Case 1, preserved layer of anterior pancreatic capsule is depicted in green colour (black arrow) highlighting the intactness of pancreas in extrapancreatic necrotising pancreatitis.

**Figure 2:**
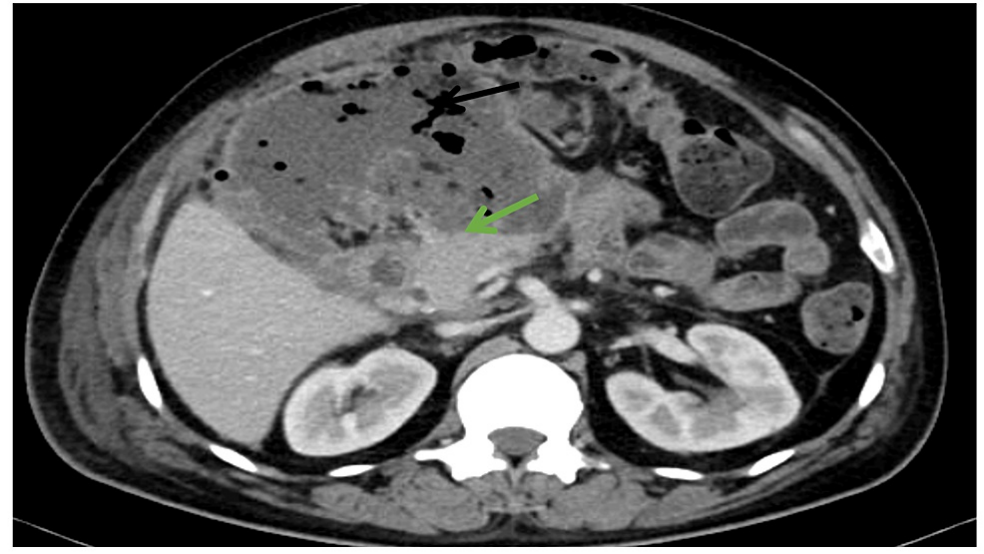
Contrast-enhanced computed tomography (CECT) image of patient in Case 1 demonstrating collection with air pockets (black arrow) adjacent to normally enhancing head of pancreas (green arrow).

**Figure 3:**
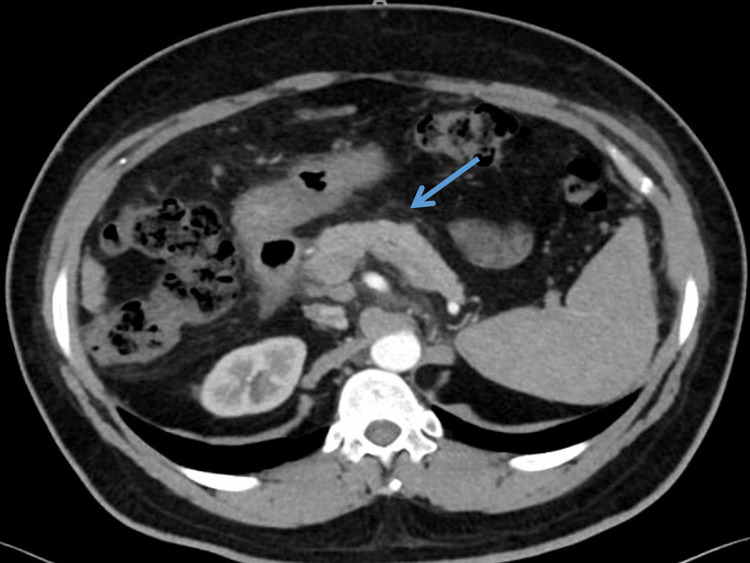
Contrast-enhanced computed tomography (CECT) abdomen showing normal pancreas (blue arrow) at six-month follow-up.

A 53-year-old male presented with sudden onset abdominal pain following a bout of binge drinking. Patient was referred to us following five weeks of initial management at another facility in view of persistent abdominal pain, febrile spikes and non-improvement of clinical status. On examination, patient was noted to have tenderness localised to epigastrium and right hypochondrium. His laboratory parameters revealed elevated total leucocyte count of 16,050 cells/mm3, elevated amylase levels of 184 IU/L and elevated lipase of 196 IU/L with normal renal and liver function parameters. Contrast-enhanced computed tomography (CECT) imaging showed normally enhancing pancreas with well-maintained lobularity on the pancreatic surface. In addition, a 13 X 11 cm extrapancreatic heterogenous septated collection with air foci was noted in the subhepatic region anterior to the stomach and duodenum. On further image processing, preserved layers of pancreatic capsule were made out and this definitively confirmed extrapancreatic presentation of pancreatitis (Figures 1, 2). Based on these findings, patient received intravenous antibiotics and underwent percutaneous catheter drainage of collection under CT guidance using 16 Fr catheter. Drain effluent was noted to be purulent initially and the quantity of daily drain output gradually decreased during further course. Patient was initially managed in the intensive care unit for three days and then was later shifted to the ward. Patient was encouraged to continue high protein enteral feeds. On the day of percutaneous drain insertion, the abdominal pain gradually subsided and the repeat total leucocyte count was 8,500 cells/mm3. On day eight post percutaneous catheter insertion, patient was discharged with advice to continue a high protein diet and for follow-up. During further follow-up, patient remained free of symptoms and the drain was removed at six weeks from the time of hospitalization after confirming a significant decrease in the size of extrapancreatic collection on imaging. At a follow-up of six months, patient was completely free from symptoms with normal blood investigations and a repeat CECT abdomen was done showing normal pancreas with complete resolution of collection (Figure 3).

Case 2

**Figure 4:**
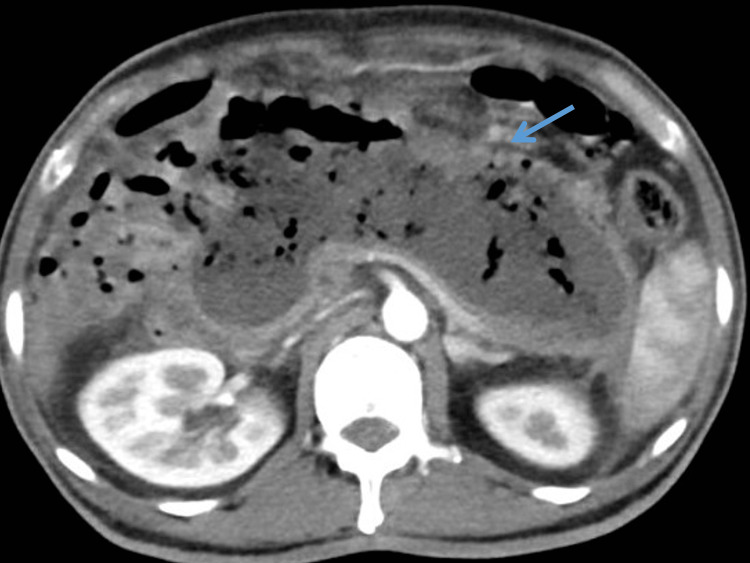
Non-contrast computed tomography (NCCT) of abdomen revealing 14 X 10 X 8 cm heterogenous collection in lesser sac with air pockets.

**Figure 5:**
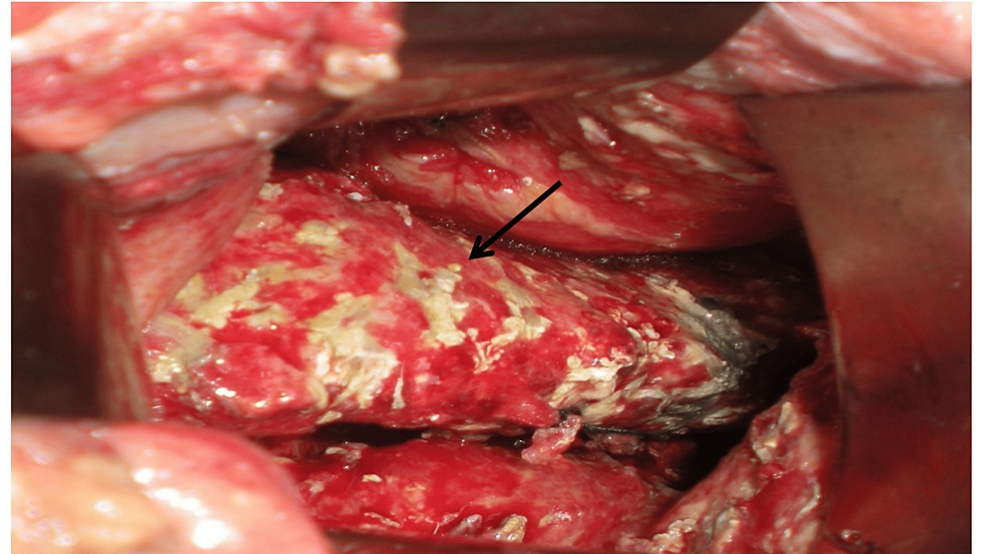
Intraoperative image showing preserved pancreas (black arrow) with necrotic flakes over the surface alone.

A 30-year-old male presented with complaints of continuous dull-aching abdominal pain, vomiting and intermittent high-grade fever for one-month duration. He received three sessions of hemodialysis for acute renal failure. He was initially managed at another facility with a provisional diagnosis of sealed duodenal perforation for more than six weeks and referred to us. At presentation, patient was febrile with tachycardia and tachypnea. On examination, decreased air entry was noted in the left hemithorax and the abdomen was distended with a tender mass in the epigastrium. Blood investigations revealed a total leucocyte count of 33,000 cells/mm3 and deranged renal parameters - blood urea of 98 mg/dl and serum creatinine of 2.8 mg/dl with normal liver function test except for low serum albumin level of 2.3 gm/dl. The serum amylase and lipase were 348 IU/L and 278 IU/L respectively. In view of respiratory distress due to confirmed massive pleural effusion on imaging, patient underwent left tube thoracostomy. The thoracostomy tube drained serous fluid of 900 ml on the day of insertion which gradually decreased. The thoracostomy amylase was normal (18 IU/L). Non-contrast computed tomography (NCCT) of abdomen revealed bulky pancreas with 14 X 10 X 8 cm heterogenous collection in lesser sac with air pockets (Figure 4). In view of deteriorating clinical condition of the patient and failed attempt of percutaneous drainage, patient was scheduled for emergency laparotomy. Intraoperatively, calcific deposits due to saponification of fat were noted over greater omentum. On exploring lesser sac, seropurulent collection with necrotic tissue was noted. Necrotic tissue was also noted around the anterior surface of entire pancreas, perisplenic area and left paracolic gutter with grossly normal appearance of pancreas in its entirety (Figure 5). Complete necrosectomy which included complete removal of necrosis was performed with placement of multiple drains for closed continuous drainage. Postoperatively, patient received closed continuous lavage with high volumes of normal saline. Initially, there was purulent discharge in the drain which gradually turned to seropurulent by day eight and then serous at day 14 postoperatively. None of the drain fluid amylase values were elevated and this ruled out possibility of pancreatic fistula. Initially, patient was managed in the intensive care unit until postoperative day three and was then shifted to the ward. Early enteral feeding on postoperative day one and early mobilization were encouraged. Tube thoracostomy was removed after confirming the reduction of pleural effusion on postoperative day five since the output was <50 ml for 48 hours. The pleural fluid and abdominal drain fluid grew Klebsiella culture and appropriate antibiotic (intravenous meropenem for five days) was given based on sensitivity pattern. General condition of patient improved gradually and the total leucocyte count, serum creatinine and blood urea normalized on postoperative day three without the further need for dialysis and was discharged with drains in situ in view of minimal purulent output. Later drains were removed during further follow-up. Absence of pancreatic fistula further highlights the extrapancreatic foci of necrosis with anatomically intact pancreas.

## Discussion

In the year 1989, Howard and Wagner attempted to study the extent of remnant pancreas following necrosectomy for acute necrotising pancreatitis patients. Among 13 patients with severe acute pancreatitis included in the study, each necrosectomy operation included complete removal of necrosis and removal of non-viable pancreas. Surprisingly, follow-up pancreatography revealed a normal or near-normal outline of the pancreatic duct among 10 out of 13 patients. Based on these findings, they have concluded that most of the necrotic tissue comprised extrapancreatic tissues in the presence of a normal viable pancreas [2]. This is the first documented evidence of extrapancreatic necrosis as a distinct entity in the presence of normal pancreas. This was further substantiated by a report by Madry et al. involving 40 patients requiring necrosectomy with predominant retroperitoneal fat necrosis and grossly intact pancreas at the conclusion of operation [3].

The presence of EPN is defined as extrapancreatic morphological changes exceeding fat stranding with complete enhancement of the pancreatic parenchyma without signs of focal or diffuse non-enhancement on the final CECT of hospitalization or before any intervention [4]. In addition to normal enhancement of pancreas, preservation of pancreatic and peripancreatic layers of tissue with intact pancreas noted on processed images of Case 1 adds significant evidence to this entity. This report intends to highlight the importance of the finding of preserved pancreatic and peripancreatic layers of tissue in defining EPN with more certainty. Sakorafas et al. studied 12 patients with extrapancreatic necrosis alone between 1983 and 1997. In addition to findings of normal enhancement pattern of pancreas on CECT, Sakorafas et al. included intraoperative evidence of lack of recognizable pancreatic parenchymal necrosis as an additional requirement for the definition of EPN. He reported that pancreas was obviously inflamed with tense parenchyma and discolored capsule, but with no evident parenchymal necrosis. This was the initial report that attempted to describe intraoperative findings of pancreas in patients with EPN. The intraoperative image findings of Case 2 in the present report correlated with the description by Sakorafas et al. [5].

In the revised Atlanta classification of acute pancreatitis, the possibility of occurrence of isolated peripancreatic necrosis with normal enhancement of pancreas was mentioned but not recognized as a distinct pathological entity of acute pancreatitis [1]. A prospective multicenter study on EPN reported that, among 639 patients with necrotising pancreatitis, almost 49% of the patients were noted to have EPN alone [4]. In a single-center study analyzing patients with acute pancreatitis over the span of two years, the incidence of EPN was reported to be 22.5% [6]. The possibility of referral bias leading to the difference in incidence of patients with EPN among the two reports cannot be ruled out.

The occurrence of perilobular fat necrosis, as one of the earliest inflammatory changes in patients with necrotising pancreatitis, probably provides an explanation for EPN. Based on the immunohistochemistry studies of Kloppel et al., perilobular fat necrosis in the presence of viable but enzymatically depleted peripheral acinar cells of lobule compared to the more centrally located acinar cells is the only theoretically possible explanation available for EPN [7]. The release of activated enzymes from peripheral acinar cells in lobules as described will trigger the necrosis of peripancreatic tissues. This finding is further substantiated by evidence of the correlation of increased levels of adipocytokines like resistin and visfatin with the presence and extent of EPN [8,9]. Another theory for EPN is based on extravasation of activated pancreatic enzymes through focal areas of parenchymal necrosis that are not detectable through available imaging. Absence of pancreatic fistulas in any of the patients with EPN in the report by Sakorafas et al. refutes the possibility of focal areas of pancreatic necrosis as a possible explanation for EPN [5].

The clinical outcomes in patients with EPN alone are found to be better than in patients with pancreatic parenchymal necrosis. An earlier report from Mayo Clinic showed less severe disease among patients with EPN with fewer long-term metabolic complications [5]. Rana et al., in their report on 48 patients with EPN, found that patients with EPN had lower frequency of pleural effusion, ascites and required fewer interventions in comparison to patients with parenchymal necrosis [6]. However, patients with EPN were not found to have lower incidence of organ failure in their findings. Bakker et al. showed that patients with EPN were found to have better outcomes in terms of decreased frequency of organ failure, need for intervention and mortality [4]. The patient of Case 1 of the present report also recovered drastically following extrapancreatic necrosectomy and closed lavage. This adds to the evidence of better outcome among patients with EPN.

## Conclusions

To conclude, extrapancreatic necrotising pancreatitis is a distinct pathological entity of acute pancreatitis. EPN can be considered as a differential diagnosis especially in doubtful cases with imaging revealing a normal pancreas. Patients with EPN alone tend to have favorable outcomes compared to patients with parenchymal necrosis. EPN is a less aggressive form of necrotising pancreatitis that needs to be defined precisely and managed accordingly.
